# Retrospective evaluation of CTV to PTV margins using CyberKnife in patients with thoracic tumors

**DOI:** 10.1120/jacmp.v15i6.4825

**Published:** 2014-11-08

**Authors:** Alejandro Floriano, Rafael García, Ramón Moreno, Alberto Sánchez‐Reyes

**Affiliations:** ^1^ Department of Medical Physics CyberKnife Unit, IMO Group Radiotherapy and Robotic Radiosurgery Center Madrid Spain; ^2^ Department of Radiotherapy Oncology CyberKnife Unit, IMO Group Radiotherapy and Robotic Radiosurgery Center Madrid Spain

**Keywords:** CyberKnife, uncertainty, thoracic treatments, CTV to PTV margin

## Abstract

The objectives of this study were to estimate global uncertainty for patients with thoracic tumors treated in our center using the CyberKnife VSI after placement of fiducial markers and to compare our findings with the standard CTV to PTV margins used to date. Datasets for 16 patients (54 fractions) treated with the CyberKnife and the Synchrony Respiratory Tracking System were analyzed retrospectively based on CT planning, tracking information, and movement data generated and saved in the logs files by the system. For each patient, we analyzed all the main uncertainty sources and assigned a value. We also calculated an expanded global uncertainty to ensure a robust estimation of global uncertainty and to enable us to determine the position of 95% of the CTV points with a 95% confidence level during treatment. Based on our estimation of global uncertainty and compared with our general margin criterion (5 mm in all three directions: superior/inferior [SI], anterior/posterior [AP], and lateral [LAT]), 100% were adequately covered in the LAT direction, as were 94% and 94% in the SI and AP directions. We retrospectively analyzed the main sources of uncertainty in the CyberKnife process patient by patient. This individualized approach enabled us to estimate margins for patients with thoracic tumors treated in our unit and compare the results with our standard 5 mm margin.

PACS number: 87.55‐x

## I. INTRODUCTION

One of the main challenges in stereotactic radiosurgery used to treat thoracic tumors is ensuring accurate delivery of radiation to targets that move with respiration. During recent decades, understanding the movement of the tumor in the thorax and its reproducibility during treatment has been an area of concern.^1^, ^2^, ^3^, ^4^, ^5^, ^6^ A variety of methods have been proposed to manage respiratory motion,^(1)^ and the margins necessary to compensate for geometric uncertainties have been reduced, depending on the technique.^7^, ^8^ The safety margin has to take into account all sources of uncertainty and is added to the clinical target volume (CTV) to reach the planning target volume (PTV). This safety margin, which is applied in order to build the PTV from the CTV, will be referred to as the PTV margin in this article.

At our center, we use the CyberKnife VSI Robotic Radiosurgery System (Accuray, Incorporated, Sunnyvale, CA), which includes the Synchrony Respiratory Tracking System v.9.6 (SRTS). This well‐documented system^3^, ^9^, ^10^, ^11^ is based on a correlation between the positions of internal and external markers that enables internal movement to be measured, compensated for, and continuously updated, so that beams can be redirected accordingly in real time.

While new imaging technologies reduce some of the uncertainty involved in setup, they are not completely free of it.^(12)^ The probability that the CTV will remain enclosed within the PTV after combining the margins for all uncertainties is particularly worrying. Small margins could result in underdosing, whereas large margins lead to more normal tissue than necessary being irradiated. A range of between 2 mm and 8 mm has been proposed and applied by different members of the CyberKnife community.^13^, ^14^, ^15^, ^16^, ^17^ At our center, we generally use 5 mm for all three directions (superior/inferior (SI), anterior/posterior (AP), and lateral (LAT)), with manual modifications, depending on the proximity of the organ at risk. Efforts are being made to investigate the different sources of uncertainty in the CyberKnife treatment process.^17^, ^18^, ^19^ With CyberKnife, final global uncertainty arises from a combination of patient‐related components (e.g., tumor movement, tumor deformation, variations in the pattern of respiration), system‐related components (i.e., predictor model version), user procedure (i.e., frequency of the X‐ray synchrony update), system geometric accuracy, and other unknown sources. The PTV margin comprises two parts: the internal margin, which is related to deformations or rotations on the CTV, and the setup margin, which takes into account uncertainties associated with the CyberKnife procedure and the way it is applied by a specific institution. Application of CyberKnife to treat a thoracic tumor requires the participation of various specialists (thoracic surgeon, radiation oncologist, medical physicist, hospital nurse, and radiation therapy technologist) to cover the whole procedure (i.e., seed implantation, CTV delimitation, planning, and treatment delivery). Differences between procedures in any of the steps could affect the associated uncertainty and the necessary safe PTV margin.

In order to evaluate treatment margins at our institution, all the main sources of uncertainty in determination of the CTV position were analyzed. The objectives of this study were to retrospectively estimate global uncertainty for the 16 patients treated in our center using SRTS and fiducial markers and compare our findings with the standard margins used to date. We compared our results against different margin setup options. Determination of the magnitude of margins is not an exact science and is, therefore, prone to assumptions; however, it can be approached systematically and analyzed according to the requirements of the individual patient.

## II. MATERIALS AND METHODS

### A. Patient database

Datasets for 16 patients treated with the CyberKnife and SRTS (54 fractions) were analyzed retrospectively based on the tracking and movement data generated and saved in the logs files by the system and on computed tomography (CT)–based planning data. Golden fiducial markers (0.5×0.8×5mm; LorcaMarín, S.A., Murcia, Spain) were placed percutaneously^20^, ^21^ and not inside the gross tumor volume (GTV) but around it in all cases, in order to reduce CT seed artifacts when delimiting CTV. At least two seeds were implanted, and we inserted an average of four seeds inside or close to the tumor. High‐resolution CT scanning was performed no fewer than six days after implantation to minimize seed migration effects.^(22)^ The CT scan was acquired with the patient's collaboration on the exhalation phase, as this is the longer phase of the respiratory cycle, in most cases. On average, treatment was started two days after CT planning. The margins used at our center were generally 5 mm in all three directions. This margin was reduced manually in cases of conflict with an organ at risk.

### B. Measurement localization procedure and data source

The general purpose of SRTS in a patient with thoracic cancers is to determine the position of the CTV points throughout the fraction in relation to the position of the robot for each beam used. Although CyberKnife makes it possible to treat patients without fiducials using the recently incorporated XSight Lung system,^(23)^ not all patients are suitable candidates for this option;^(24)^ therefore, placement of fiducial markers is necessary in some cases. The center of mass for the CT scan $\left({\text{CoM}}_{\text{CT}}\right)$ of the fiducial configuration is the initial information tracked. The system builds a correlation model by correlating the internal markers and the infrared external markers placed on the patient's chest during different phases of the respiratory cycle.^(3)^ As the position of the linear accelerator needs 115 ms to be adjusted, a predictor model is used during treatment.^(25)^ Using a global positioning system (GPS) analogy, the procedure to determine a single point inside the GTV position in relation to the delivered beam can be split into four different steps:

1 Step 1. Relative positioning between the ${\text{CoM}}_{\text{CT}}$ and arbitrary positioning $\left(X_{\text{ti}},Y_{\text{ti}},Z_{\text{ti}}\right)$ in the CTV.
2 Step 2. Based on the seed configuration during the treatment, variation of the position of the center of mass obtained from the live images during treatment $\left({\text{CoM}}_{\text{LIVE}}\right)$ in relation to the ${\text{CoM}}_{\text{CT}}$.
3 Step 3. Correlation between ${\text{CoM}}_{\text{LIVE}}$ and the external markers.
4 Step 4. Association between the respiratory model and the predictor model.

**Figure 1:**
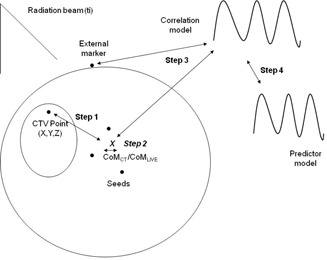
Process for determining the position of a CTV point using SRTS. Each step has an associated standard uncertainty. Step 1: CTV‐seed deformation $\left(e_{\text{deformation}}\right)$. Step 2: seed configuration variation $\left(e_{\text{seeds}}\right)$. Step 3: correlation model $\left(e_{\text{correlation}}\right)$. Step 4: predictor model $\left(e_{\text{prediction}}\right)$.

**Figure 2:**
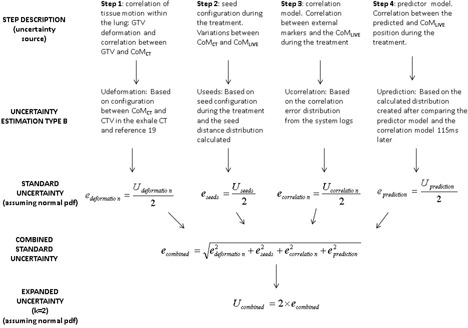
Process for determining the position of a CTV point using SRTS. Each step has an associated standard uncertainty. Step 1: CTV‐seed deformation $\left(e_{\text{deformation}}\right)$. Step 2: seed configuration variation $\left(e_{\text{seeds}}\right)$. Step 3: correlation model $\left(e_{\text{correlation}}\right)$. Step 4: predictor model $\left(e_{\text{prediction}}\right)$.

Every step taken to track the CTV position is a source of uncertainty that contributes to the overall uncertainty. In this general description, and for purposes of simplification, we focus on a single point and explain the whole process in order to determine one CTV point at a defined time $\left(X_{\text{ti}},Y_{\text{ti}},Z_{\text{ti}},t_{i}\right)$. We retrospectively analyzed all the main steps and assigned an uncertainty value to specific steps for each patient. For a specific time point $t_{i}$, we analyzed the uncertainty for the four main steps in order to determine the position $\left(X_{\text{ti}},Y_{\text{ti}},Z_{\text{ti}}\right)$ in relation to the robot position for a beam (Figs. 1 and 2). In order to estimate the uncertainty associated with each step, we used the logs files generated by the CyberKnife, including the AlgorithmImaging. log, which contains 3D information about the seed configuration for each live image. Previous literature results were used in those steps where uncertainty could not be estimated based only on our dataset.

### C. Estimating measurement uncertainty

In order to establish a combined expanded uncertainty for the whole process, an individual uncertainty was assigned to each step. We evaluated all sources of uncertainty independently. Although we assumed that this approach did not reflect the real situation (i.e., seed deformation could have an effect on the accuracy of the correlation model), we considered it a conservative assumption about uncertainties in this process. All sources were considered Type B uncertainties because, in most cases, we used parameters that were only indirectly associated with the uncertainty of the step studied or because we were not able to obtain statistical information; in such cases, uncertainty was assigned based on previous literature. When simplifications were made, we were always conservative in our analysis in order not to underestimate the final combined uncertainty for the process.

Each target movement direction was also analyzed independently. For an individual patient (j=1 to 16), we estimated the uncertainty (${U_{m}}^{\text{ij}}$) and standard uncertainty (${e_{m}}^{\text{ij}}$) for each step (for m=1 to 4) and each movement direction (for i=1 to 3) and calculated the combined standard uncertainty ($e^{\text{ij}}$) as follows:

(1) $$e_{\text{combined}}^{\text{ij}}=\sqrt{e_{\text{deformation}}^{\text{ij}2}+e_{\text{seeds}}^{\text{ij}2}+e_{\text{correlation}}^{\text{ij}2}+e_{\text{prediction}}^{\text{ij}2}}$$

An expanded uncertainty for each axis movement for the whole process with a 95% confidence level was defined as follows:

(2) $$U_{\text{combined}}^{\text{ij}}={\text{ke}}_{\text{combined}}^{\text{ij}}$$

with k=2 (for i=1 to 3).

Following these steps, we were able to estimate the uncertainty associated with the global process of determining the position of 95% of the CTV points ($X_{\text{ti}},Y_{\text{ti}},Z_{\text{ti}}$), with a 95% confidence level for a beam at any time $t_{i}$. Based on geometrical considerations only, we believe this is an estimation of the individual PTV margins.

### D. Sources of uncertainty

#### D.1 ${\text{CoM}}_{\text{CT}}$ position related to any CTV point position

Strictly speaking, the system does not follow the target; rather, it follows the ${\text{CoM}}_{\text{CT}}$ of the fiducial configuration implanted some days before treatment. Manufacturer recommendations on the distance between seeds and the number of seeds were followed during implantation in order to ensure accurate delivery of treatment.^(26)^ The degree to which the motion of an implanted thoracic fiducial configuration correlates with the motion of a tumor typically deteriorates when the distance between the fiducials and the tumor increases.^(19)^ This problem is heavily dependent on individual patient features (e.g., internal target deformation, target position, movement of the thorax, and fiducial configuration)^19^, ^27^, ^28^ and is one of the main sources of uncertainty. In addition, ${\text{CoM}}_{\text{CT}}$ position is very difficult to modify, as it is not possible to change a configuration once the seed has been implanted. Since this source of uncertainty could not be calculated directly and retrospectively, we assigned uncertainties based on Smith et al.,^(19)^ taking the ${\text{CoM}}_{\text{CT}}$ position in relation to the target for each patient as an input parameter for the uncertainty value selection. Smith and colleagues define the correlation radius as the maximum radius at which the magnitude of the vector motion of 95% of the voxel surrounding a seed point correlated to within 3 mm of the motion of the seed voxel, with a mean correlation radius for the tumor area of 3.1 cm. The authors also plot the correlation error as a function of the distance from the tumor centroid (Smith et al. Fig. 5^(19)^). Based on this information, and depending on the position between the ${\text{CoM}}_{\text{CT}}$ with respect to the CTV center of mass and to the closest border analyzed from the exhale CT for each patient measured in the planning system, we assigned the uncertainty related to Step 1 (independently of the coordinate direction) for each patient, j, and coordinate, i, as follows:

1 If ${\text{CoM}}_{\text{CT}}$ was inside the CTV, ${U^{\text{ij}}}_{\text{deformation}}=2.1\;\text{mm}$ (based on Smith et al. Fig. 5^(19)^);
2 If ${\text{CoM}}_{\text{CT}}$ was outside the CTV, but the distance from the closest border (measured in the CT as the smallest distance in the three planes) was $<30\;\text{mm},{U^{\text{ij}}}_{\text{deformation}}=3.0\;\text{mm}$.

This uncertainty simultaneously takes into account deformations and rotations of the tumor inside the CTV and variations in the movement of the ${\text{CoM}}_{\text{CT}}$ and any CTV point during the treatment.

As all the patients had one of these two options, no extra conditions were added. To calculate the standard uncertainty, a normal probability density function (PDF) distribution was assumed, so that ${e^{\text{ij}}}_{\text{deformation}}={U^{\text{ij}}}_{\text{deformation}}/2$. Table 1 shows the configuration related to center of the CTV and the closest CTV border for the 16 patients analyzed. ${\text{CoM}}_{\text{CT}}$ was used as the input value for determination of this uncertainty step.

**Table 1:** Distances in the fiducial configuration. Association with the CTV center and nearest border. Positive and negative values represent ${\text{CoM}}_{\text{CT}}$ outside and inside the CTV, respectively.

*Patient Number*	*Number of Seeds Introduced*	*Number of Seeds for Tracking*	*Distance From Center of CTV to* ${\text{CoM}}_{\text{CT}}$ *(mm)*	*Distance From Border of CTV to* ${\text{CoM}}_{\text{CT}}$ *(mm)*
1	4	4	10.7	1.4
2	3	3	24.6	−4.2
3	4	4	13.4	3.2
4	4	4	18.2	5.0
5	3	3	10.9	−0.9
6	4	3	22.9	12.2
7	3	2	15.0	−2.3
8	4	4	5.6	−1.0
9	4	4	13.3	8.6
10	2	2	6.3	−2.6
11	3	2	19.9	15.2
12	4	3	11.5	−0.6
13	3	2	25.2	17.2
14	3	2	20.0	17.0
15	2	2	29.0	21.0
16	4	3	9.5	2.3

#### D.2 ${\text{CoM}}_{\text{LIVE}}$ vs. ${\text{CoM}}_{\text{CT}}$ seed configuration

The seed configuration obtained from the CT scan was used as the reference to define the ${\text{CoM}}_{\text{CT}}$. This configuration is not rigid, and various small deformations may arise during treatment, depending on the positions of the fiducial markers and their independent movement inside the thorax. In cases where migrations or high deformation values were detected after the CT, the problematic seed was disabled for tracking.

During the fraction, CyberKnife reports the solid rigid error (${\text{SR}}_{\text{error}}$), which is defined as the maximum absolute difference in the distance between each pair of seeds between the CT position and the LIVE position, as follows:

(3) $${\text{SR}}_{\text{error}}=\left|\text{max}(d_{i\text{jCT}}-d_{\text{ijLIVE}})\right|$$

This parameter is to a certain extent associated with the deformation of the seed configuration and the deviation of ${\text{CoM}}_{\text{LIVE}}$ from ${\text{CoM}}_{\text{CT}}$, and, consequently, with the accuracy of the ${\text{CoM}}_{\text{LIVE}}$ position and its relation to the CTV position points. The standard limit for ${\text{SR}}_{\text{error}}$ is 1.5 mm; the system cannot treat at values higher than 5 mm. In our center, we evaluated each situation individually. In the case of a large ${\text{SR}}_{\text{error}}(>3\;\text{mm})$, the migrated or uncorrelated seed was removed from the tracking.

${\text{CoM}}_{\text{LIVE}}$ will vary around ${\text{CoM}}_{\text{CT}}$ during treatment, and this variation can be estimated from seed data. The association between the ${\text{SR}}_{\text{error}}$ and the ${\text{CoM}}_{\text{LIVE}}$ position in relation to ${\text{CoM}}_{\text{CT}}$ is only an approximation, as the determination of the ${\text{CoM}}_{\text{LIVE}}$ and its variation from the ${\text{CoM}}_{\text{CT}}$ is a complex geometrical problem. In order to determine as accurately as possible the uncertainty assigned to Step 2, we used AlgorithmImaging.log to obtain the distance positions between all the seeds in a live image and compared the results with the CT scan distance each time a live image was acquired (with positive values for seeds separating at a specific time and negative values for seeds closing at a specific time in relation to the CT scan). The distribution of the difference in distances between the seeds was calculated for each fraction and patient. We used this distribution to estimate the uncertainty associated with Step 2. We assumed that the maximum error in the ${\text{CoM}}_{\text{LIVE}}$ localization related to ${\text{CoM}}_{\text{CT}}$, regardless of the number of seeds placed, would be half of the values obtained (as happens in the simplest case, with only two fiducials, as the variation in distance between seeds equals half of the variation of the ${\text{CoM}}_{\text{LIVE}}$ around ${\text{CoM}}_{\text{CT}}$). The same global value was assigned to each movement direction; therefore, for each patient, j, and direction, i, the $U_{\text{seeds}}$ assigned was

(4) $$U_{\text{seeds}}^{\text{ij}}=\frac{\left|\mu^{j}\right|+2\sigma^{j}}{2}$$

with |μ| as the absolute mean value and σ as the standard deviation from the distribution of the difference in distance between the seeds. The standard associated uncertainty assuming normal PDF was $e_{\text{seeds}}=U_{\text{seeds}}/2$.

#### D.3 Correlation model

A correlation model was created to relate the signal position of the external markers to the internal fiducial ${\text{CoM}}_{\text{LIVE}}$. The subsystem has been widely described elsewhere.^(3)^ In our center, we update the correlation model by taking X‐ray images every minute. A parameter obtained from the CyberKnife is used to estimate the standard uncertainty directly from the probabilistic distribution. The correlation error reported by the system after each live image was used. This parameter measures differences between the modeled point position and the actual position.^(7)^ If the correlation model error exceeds 2 mm, we decrease the X‐ray acquisition time before continuing irradiation; for values higher than 3 mm appearing three times consecutively, we rebuild the model. Both the values used to create each model before irradiation and those values that led us to cancel an existing model and rebuild a new one were excluded from the analysis. This updating procedure is specific to our institution, and the procedures used at other institutions may vary. A correlation error distribution was obtained once the fraction was complete. For each direction, i, and patient, j, the uncertainty assigned was

(5) $$U_{\text{correlation}}^{\text{ij}}=\left|\mu^{\text{ij}}\right|+2\sigma^{\text{ij}}$$

with |μ| as the absolute mean value and σ as the standard deviation from the correlation error distribution. The standard associated uncertainty assuming normal PDF was ${e^{\text{ij}}}_{\text{correlation}}={U^{\text{ij}}}_{\text{correlation}}/2$.

Although this step is not independent of Step 2, we considered them independent and conservatively assumed that all the uncertainty for this step was due to changes in the respiratory cycle from the external markers, variations in patient respiratory cycles, correlation between skin and internal fiducials, and unknown sources.^4^, ^5^, ^6^, ^29^, ^30^ We assumed that the variation in the seed configuration during treatment had no effect on the correlation model.

#### D.4 Prediction model

The prediction model used in the CyberKnife has been investigated by several authors.^17^, ^18^ We carried out the same procedure from Step 3 for each patient, although we used the error distribution calculated by comparing the predictor model with the correlation model position 115 ms later. From this error distribution we estimated the uncertainty, depending on the coordinate direction. All the data saved from the prediction log file were used in the analysis, independently of radiation beam status. For each direction, i, and patient j, we defined and calculated

(6) $$U_{\text{prediction}}^{\text{ij}}=\left|\mu^{\text{ij}}\right|+2\sigma^{\text{ij}}$$

with |μ| as the absolute mean value and σ as the standard deviation from the comparison between the correlated error distribution and the predicted error distribution. The standard associated uncertainty assuming a normal PDF was ${e^{\text{ij}}}_{\text{prediction}}={U^{\text{ij}}}_{\text{prediction}}/2$.

### E. ${\text{CoM}}_{\text{LIVE}}$ peak‐to‐trough distance: range of movement

A range of movement for each treatment session was calculated for each patient based on the correlation model generated by CyberKnife for each fraction. In order to obtain a straightforward value from the logs files to characterize the global movement fraction by fraction, no previously proposed exclusion criterion was used,^(6)^ and a peak‐to‐trough amplitude was calculated for each correlation model time recorded. We were thus able to calculate two parameters for each session: the mean peak‐to‐trough amplitude for each axis during the treatment ($R_{m}$), and the 95% quartile mean‐to‐trough amplitude ($R_{95\%}$), which enabled us to reject variations resulting from unexpected deep inspirations or changes due to couch movement during treatment. The median and mean values excluding the smaller 5% and higher 95% peak‐to‐trough amplitude were also calculated. The range of movement thus obtained did not take into account the intrafractional variability of tumor motion baseline.

## III. RESULTS

### A. ${\text{CoM}}_{\text{LIVE}}$ range of movement

Table 2 shows the mean and the standard deviation from the $R_{m}$ and $R_{95\%}$ values calculated for each patient based on the individual treatment fraction. Although determination of the range of movement was not a main objective of this article, our results shows that movement was generally greater in the SI direction than in the other directions and in tumors generally located in the lower lung, although some unexpected results were obtained (for Patient number 13, the tumor was in the upper lung, with a higher movement in the AP direction). Indeed, since both parameters seem to have a small standard deviation, a patient parameter can be obtained to estimate the range of movement. The results seem to be similar to those obtained by previous authors for the range of movement.^4^, ^6^ The median and mean values excluding the 5% smaller and 95% higher values were similar to the $R_{m}$ in each fraction, with differences lower than 1 mm in most situations (results not shown).

**Table 2:** Mean and standard deviation (number of fractions between 3 and 5) for the $R_{m}$ and $R_{95\%}$ result for each patient and direction.

*Patient Nnumber*	${R_{m}}^{X–\text{SI}}$ *(mm)*	${R_{m}}^{Y–\text{LAT}}$ *(mm)*	${R_{m}}^{Z–\text{AP}}$ *(mm)*	${R_{95\%}}^{X–\text{SI}}$ *(mm)*	${R_{95\%}}^{Y–\text{LAT}}$ *(mm)*	${R_{95\%}}^{Z–\text{AP}}$ *(mm)*
1	17.0±0.4	1.6±0.5	1.6±0.2	22.6±1.9	2.2±0.5	2.3±0.1
2	0.3±0.1	0.3±0.1	0.2±0.1	0.7±0.1	0.6±0.2	0.5±0.1
3	4.1±0.4	0.4±0.2	1.7±0.1	5.1±0.7	1.1±0.4	2.4±0.1
4	0.2±0.1	0.3±0.1	0.4±0.1	0.5±0.3	1.0±0.4	0.9±0.3
5	13.5±0.3	1.0±0.1	1.6±0.4	18.5±0.9	1.9±0.1	3.0±0.2
6	4.4±0.6	0.4±0.2	0.8±0.3	6.3±0.2	1.2±0.3	2.1±0.4
7	8.6±0.7	2.9±0.4	2.0±0.4	11.3±0.9	4.0±0.7	2.8±0.3
8	3.9±1.4	0.6±0.7	4.0±0.4	7.9±0.9	0.6±0.7	6.2±0.9
9	2.3±0.5	1.5±0.3	3.3±0.3	3.6±0.5	2.5±0.2	4.6±0.5
10	8.1±0.3	1.0±0.4	3.2±1.2	10.2±0.6	1.6±0.4	4.4±1.6
11	14.0±0.6	2.0±0.1	0.9±0.2	24.0±4.1	4.1±1.2	2.1±0.5
12	1.4±0.2	0.5±0.1	2.2±0.5	2.7±0.5	1.2±0.3	4.3±1.4
13	7.1±2.3	1.6±1.0	9.3±3.6	10.9±2.6	3.0±1.0	14.4±4.0
14	1.5±0.2	4.3±0.1	1.7±0.4	2.7±0.2	6.4±1.0	3.5±0.7
15	10.6±0.9	0.8±0.3	1.2±0.3	15.1±1.1	1.7±0.3	2.1±0.4
16	3.7±0.3	0.6±0.2	2.8±0.9	5.1±0.3	1.0±0.1	4.3±1.2

### B. Individual step uncertainties

**Figure 3:**
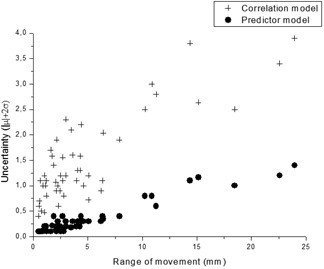
$U_{\text{correlation}}$ and $U_{\text{prediction}}\;(|\mu |+2\sigma )$ uncertainties as a function of the range of movement ($R_{95\%}$) in each direction.

Table 3 shows the results for each patient of the uncertainty calculated and assigned for each step. In the case of the $U_{\text{deformation}}$, assignation of uncertainty depended on the distances shown in Table 1. The same global uncertainty value was assigned for the three movement axes for $U_{\text{deformation}}$ and $U_{\text{seeds}}$. The $U_{\text{correlation}}$ and $U_{\text{prediction}}$ were calculated according to the movement axis and plotted against $R_{95\%}$ (Fig. 3). From the figure, a positive correlation was established between the uncertainties estimated for Steps 3 and 4 and $R_{95\%}$. A similar pattern was obtained when $R_{m}$ was used (not shown). In the case of the correlation errors obtained, the mean correlation error for all the fractions and patients was 1.5±0.8mm, which is comparable to those of a previous analysis with 14 treatments from Kilby et al.^(31)^ with values of 1.4±1.0mm. Predictor error was lower than previously published,^(18)^ probably as a result of changes introduced in the most recent version, in which the hybrid prediction algorithm was applied.^(25)^

**Table 3:** Step 1 ($\text{CTV}/{\text{CoM}}_{\text{CT}}\;\text{deformation}$) and Step 2 (${\text{CoM}}_{\text{CT}}/{\text{CoM}}_{\text{LIVE}}\;\text{variation}$) assigned uncertainties. The values are applied for each coordinate independently. Step 3 (correlation model) and Step 4 (prediction model) show assignation of uncertainties for each coordinate.

*Patient Number*	$U_{\text{deformation}}$ *(mm)*	$U_{\text{seeds}}$ *(mm)*	${U^{X–\text{SI}}}_{\text{correlation}}$ *(mm)*	${U^{Y–\text{LAT}}}_{\text{correlation}}$ *(mm)*	${U^{Z–\text{AP}}}_{\text{correlation}}$ *(mm)*	${U^{X–\text{SI}}}_{\text{prediction}}$ *(mm)*	${U^{Y–\text{LAT}}}_{\text{prediction}}$ *(mm)*	${U^{Z–\text{AP}}}_{\text{prediction}}$ *(mm)*
1	3.0	0.9±0.2	3.4±0.3	1.0±0.2	0.6±0.1	1.2±0.2	0.3±0.1	0.2±0.1
2	2.1	0.9±0.3	0.5±0.2	0.7±0.2	0.4±0.2	0.1±0.1	0.1±0.1	0.1±0.1
3	3.0	1.2±0.2	1.2±0.2	1.0±0.2	0.9±0.1	0.3±0.1	0.2±0.1	0.2±0.1
4	3.0	1.0±0.4	0.6±0.1	1.2±0.2	1.0±0.1	0.1±0.1	0.2±0.1	0.1±0.1
5	2.1	1.0±0.2	2.5±0.3	1.4±0.1	2.3±0.3	1.0±0.1	0.4±0.1	0.2±0.1
6	3.0	1.8±0.2	1.1±0.1	1.1±0.5	1.9±0.3	0.4±0.1	0.2±0.1	0.2±0.1
7	2.1	2.0±1.0	2.8±0.3	1.1±0.3	0.8±0.1	0.6±0.1	0.2±0.1	0.1±0.1
8	2.1	1.0±0.1	1.9±0.5	1.1±0.3	0.9±0.2	0.4±0.1	0.1±0.1	0.3±0.2
9	3.0	0.6±0.1	1.6±0.3	1.2±0.3	1.0±0.2	0.3±0.1	0.1±0.1	0.3±0.2
10	2.1	1.2±0.3	2.5±0.7	1.7±0.3	2.2±0.2	0.8±0.1	0.1±0.1	0.4±0.1
11	3.0	0.5±0.1	3.9±0.1	1.3±0.3	0.9±0.1	1.4±0.5	0.3±0.1	0.1±0.1
12	2.1	1.6±0.2	1.1±0.5	0.8±0.3	1.3±0.3	0.4±0.4	0.1±0.1	0.2±0.1
13	3.0	1.5±0.3	3.0±0.2	1.2±0.4	3.8±0.1	0.8±0.3	0.3±0.1	1.1±0.5
14	3.0	1.0±0.5	1.6±0.2	2.0±0.2	2.1±0.2	0.2±0.1	0.3±0.1	0.2±0.1
15	3.0	1.2±0.3	2.6±0.2	1.6±0.4	1.1±0.2	1.2±0.1	0.2±0.1	0.1±0.1
16	3.0	1.3±0.3	0.7±0.1	0.5±0.1	1.6±0.1	0.3±0.1	0.2±0.1	0.3±0.1

### C. Global uncertainty

Table 4 shows the global uncertainty (k=2) according to the movement axis. These results would represent an estimation of global uncertainty to determine the position of 95% of the CTV points with a 95% confidence level during treatment; therefore, they were compared with the margins applied for each patient. Table 5 shows the percentage of patients passing the different possible margins (from 2 mm to 6 mm). Based on our estimation of global uncertainty and compared with our general margin criterion (5 mm in all three directions), 100% were adequately covered in the LAT direction, as were 94% and 94% in the SI and AP directions.

**Table 4:** Global estimated uncertainty for each coordinate based on the four steps considered as independent and assuming a normal Gaussian PDF.

*Patient Number*	$U^{X–\text{SI}}$ *(mm)*	$U^{Y–\text{LAT}}$ *(mm)*	$U^{Z–\text{AP}}$ *(mm)*
1	4.8	3.3	3.2
2	2.3	2.4	2.3
3	3.4	3.4	3.4
4	3.2	3.4	3.3
5	3.6	2.7	3.3
6	3.7	3.7	4.0
7	4.1	3.1	3.0
8	3.0	2.6	2.5
9	3.5	3.3	3.2
10	3.5	2.9	3.3
11	5.1	3.3	3.2
12	2.9	2.8	3.0
13	4.6	3.6	5.2
14	3.6	3.7	3.8
15	4.2	3.6	3.5
16	3.4	3.4	3.7

**Table 5:** Percentage of patients fulfilling the margin criterion based on the global uncertainty calculated.

*Margin*	*X‐SI*	*Y‐LAT*	*Z‐AP*
2 mm	0%	0%	0%
3 mm	12%	25%	12%
4 mm	68%	100%	93%
5 mm	94%	100%	94%
6 mm	100%	100%	100%

## IV. DISCUSSION

Individualizing margins for a thoracic tumor is problematic, and margin selection is generally based on standard values for each institution. To date, we have used a 5 mm margin, with reductions in cases where it was considered necessary owing to the proximity of the organ at risk. In any case, margins should be analyzed patient by patient, based on the best available knowledge of the process.^(1)^ Since much of the useful information obtained by applying this approach is only available after treatment, our preferred option was the general standard margin, as reported in the literature.^(18)^ In order to validate the general standard margin in our institution, we performed a retrospective patient‐by‐patient analysis of the movement of each lesion and the associated uncertainties.

We investigated the movement of the lesion with the movement of ${\text{CoM}}_{\text{LIVE}}$ during each treatment fraction. Mean cycle‐to‐cycle motion amplitude ± standard deviation was 6.3±5.2mm (SI direction), 1.2±1.0mm (LAT direction), and 2.3±2.1mm (AP direction) — that is, close to the means of 6.0±4.6mm and 5.0±1.6mm reported by Chan et al.^(4)^ and Suh et al.,^(6)^ respectively, in the craniocaudal axis. Since interfraction variation of $R_{m}$ and $R_{95\%}$ is under 2 mm in most patients, it seems that, although the movement for a thoracic lesion changes during a fraction, some parameters for each fraction remain stable, with a small standard deviation.

Tumor deformations, correlations with surrounding tissue, and fiducial variation in CoM compared to ${\text{CoM}}_{\text{Tumor}}$ have been studied by other authors.^19^, ^27^, ^28^, ^29^ In our split methodology, these data would be useful for determination of the uncertainties associated with Steps 1 and 2. Ueki et al.^(27)^ recently showed the variation between the respective CoM for the tumors and the implanted markers to be under 2.5 mm using 4D CT for 15 patients and found that interfractional variation was much more important than intrafractional variation. These results are comparable with those of our patient‐by‐patient analysis based on the seed configuration and proximity of the CoM to the GTV and the deformability of the seed configuration during treatment.

The correlation we found between the ${\text{CoM}}_{\text{LIVE}}$ movement range and correlation/prediction errors could prove useful in the future. Similar results were obtained by Hoogeman et al.^(18)^ who used different CyberKnife versions. In the case of the prediction method, these authors calculated both the error for the clinical algorithm used at the time (historic, 192.5 ms) and a simulation of the new algorithm implemented (hybrid, 115 ms), observing a reduction in the slope (plotted from the standard deviation error and the range of tumor motion) from 0.12 to 0.06. In our case, plotting the standard deviation prediction model against $R_{m}$ (not shown) would result in a slope of 0.04, which is similar to the simulated results reported by Hoogeman et al.^(18)^

Our patient‐by‐patient global uncertainties margin is consistent with the results obtained using a different methodology, namely, global population margins. Sawkey et al.^(12)^ compared combined uncertainties for different motion management strategies. In their simulation of tracking with a latency of 100 ms, which is near the values for the CyberKnife system, they show margins ranging between 2.2 and 4.9 mm, with a mean of 3.1 mm, although they used a rigid CTV that did not rotate in their analysis. Pepin et al.^(17)^ showed global results based on the modeler+predictor+machine uncertainties summed for their population, namely, aggregated errors of 6.9 mm, 4.6 mm, and 3.5 mm in the SI, AP, and LAT directions, respectively; however, no uncertainty related to GTV deformation or seed movement in relation to GTV movement was taken into account. Lu et al.^(28)^ analyzed the GTV internal deformation for 17 patients and the bibliography for the uncertainty in tracking and fiducial migration reported global margins of between 4.1 mm and 7 mm for the quadratic sum and arithmetic sum, respectively. George et al.^(32)^ performed a population margin analysis based on the formula by Stroom et al.^(33)^ and reported values for real‐time tracking of between 0.5 mm in the “optimistic case” and 8.2 mm in the “realistic case”, where the contribution of both 3 mm systematic and random error from other sources was taken into account for the second case. The results obtained using our methodology are compatible with the results of the abovementioned authors in all cases when the four main uncertainty sources are taken into account patient by patient.

The benefit of the 4D CT scan used for planning purposes in CyberKnife is being studied elsewhere.^34^, ^35^ As our institution does not have 4D CT, a collaborative deep inspiration/deep expiration CT scan could prove useful when attempting to estimate an upper limit to the amount and direction of tumor motion that could be useful when analyzing the necessary margins. This motion is not predictable by location,^5^, ^36^ as we observed in specific cases. Although intrafraction motion has been demonstrated,^4^, ^6^, ^27^ we observed that $R_{m}$ and $R_{95\%}$ were stable estimators during a treatment for each patient. The collaborative CT scan could be used as a basis to obtain a general “feel” for the $R_{95\%}$ peak‐to‐peak amplitude of breathing motion for a specific patient, to eliminate obvious seeds not correlated with GTV movement, and to determine an estimated upper limit for each step by basing Step 1 on ${\text{CoM}}_{\text{CT}}$ and tumor position distance and Steps 3 and 4 in the association between uncertainties and range movement (Fig. 3). For Step 2, depending on the tumor position and number of seeds available, a $U_{\text{seed}}$ value of between 1 mm and 2 mm could be selected. This previous uncertainty analysis would prove useful in cases where several disadvantageous situations occur in the same patient (e.g., ${\text{CoM}}_{\text{CT}}$ far from the GTV, high range of movement, and small number of seeds). Based on these preliminary results, we decided to expand the margin to 6 mm in situations where the ${\text{CoM}}_{\text{CT}}$ was located outside the CTV and where the breathing motion amplitude in the CT scan is around ≥15mm (as was the case in the two patients with a calculated uncertainty >5mm). However, our findings will be updated continuously with the inclusion of new patients. In our study, the ${\text{CoM}}_{\text{CT}}$ was not outside the 30 mm CTV closest border limit. In such cases, a careful analysis should be carried out.

Our study is limited by the fact that the results are based on data from only 16 patients. However, data from the steps described above would enable us to update our model systematically in order to verify or reject it. Comparison of our results with the 5 mm standard margin showed that 100% were adequately covered in the LAT direction, as were 94% and 94% in the SI and AP directions, with a margin ranging between 2.3 mm and 5.2 mm. Given that the assumptions we made to estimate the uncertainties associated with each step could affect the global results, we intend to perform further studies to obtain more robust data. However, in the meantime, based on geometrical considerations, we consider that a 5 mm isotropic margin is safe enough in most cases.

Our study was based on the geometrical relationship between a CTV point and a beam in a patient‐by‐patient analysis. We decided not to consider any uncertainty related to global delivery and accuracy based on phantom measurements end‐to‐end (E2E).^(9)^ There are two reasons for this decision. On the one hand, we think that the E2E test would include some of the errors included in the patient‐by‐patient study (correlation and prediction model for the phantom); therefore, if the E2E test is kept under 1 mm with a robotic manipulator precision of 0.12 mm,^(9)^ we consider that this need not be taken into account. However, if the E2E is to be taken into account, we should add a $U_{\text{tracking}}=0.7\pm 0.3\;\text{mm}$ (n=20, from our database measurement E2E results). Adding this value in a quadratic sum would add between 0.1–0.2 mm to the combined global uncertainties estimated, depending on the patient. On the other hand, we based our study only on geometrical uncertainties and not on CTV coverage; the E2E accuracy is a mixture of both terms, as radiation is delivered. We used a purely geometrical approach because we believe that the PTV margin definition used by van Herk et al.^(37)^ is not directly applicable at this time in the CyberKnife system (“The PTV is a geometrical concept … taking into consideration the net effect of all the possible geometrical variations and inaccuracies in order to ensure that the prescribed dose is actually absorbed in the CTV”), as the treatment planning algorithm used to date has a much more significant impact on the correct dose for tumor coverage^38^, ^39^, ^40^ and real optimal dose prescription.^(41)^ Therefore, the clinical implication of the margin selected is unclear.

Lastly, the CyberKnife system enables treatment of thoracic tumors without inserting seeds using the XSight Lung System. The effect of this approach on the calculation of uncertainties would be double: it would eliminate only part of the Step 1 uncertainty (by maintaining the inner deformation of the tumor) and it would completely eliminate the Step 2‐assigned uncertainty (the seed deformation during the treatment), although it would be necessary to add a new uncertainty related to the accuracy of the tracking patient by patient, as not all the patients would be candidates for tracking to follow the lesions. Efforts are being made to make this selection criterion as objective as possible.^(24)^

## V. CONCLUSIONS

We retrospectively analyzed the main sources of uncertainty in the CyberKnife process patient by patient. This individualized approach enabled us to estimate margins for patients with thoracic tumors treated in our unit and to compare the results with our standard 5 mm margins. We found that this is generally a safe geometric margin. A procedure for pre‐estimating margins patient by patient has been set up, and methods to improve our knowledge for each associated uncertainty step are desirable. We intend to update the data reported here with a greater number of patients and thus generate more robust results.

## ACKNOWLEDGMENTS

The authors thank Mr. Raffaele Meroni for his helpful revision and suggestions and Mr. Thomas O'Boyle for his help in preparing the manuscript. I would also like to thank the referees for their helpful comments, which have substantially improved this paper.

## Supporting information

Supplementary Material
