# Supplementary figures and images for: Retrospective evaluation of CTV to PTV margins using CyberKnife in patients with thoracic tumors
Source: J Appl Clin Med Phys. 2014 Nov 8;15(6):59–72. doi: 10.1120/jacmp.v15i6.4825 (PMC5711121; doi:10.1120/jacmp.v15i6.4825)

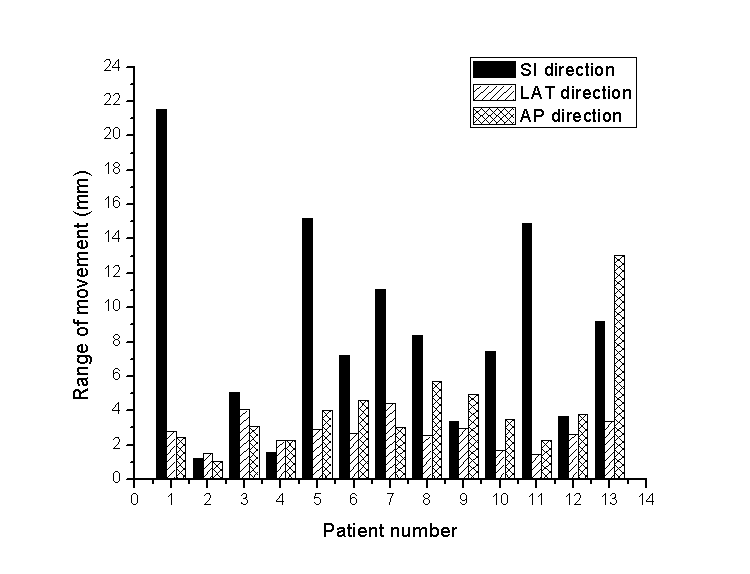

Supplement: Supplementary file 1 — Supplementary Material [file ACM2-15-059-s001.gif]
